# Follow-up study to explore the relationship between Neutrophil to lymphocyte ratio and impaired fasting glucose—using the group-based trajectory modeling

**DOI:** 10.1038/s41598-024-64701-5

**Published:** 2024-06-18

**Authors:** Xuekui Liu, Gangshan Peng, Ran Liu, Xiu Zang, Caiyan Zou, Haojie Sun, Qian Zhu, Houfa Geng, Jun Liang

**Affiliations:** 1 Department of Endocrinology, Xuzhou Central Hospital, Xuzhou Medical University Affiliated Xuzhou Clinical Collage, Xuzhou, Jiangsu China; 2 Xuzhou Medical University, Xuzhou, Jiangsu China; 3 Quanshan Taishan Community Hospital, Xuzhou, Jiangsu China

**Keywords:** Neutrophil to lymphocyte ratio, Impaired fasting glucose, Body mass index, Group-Based Trajectory Modeling, Endocrinology, Medical research

## Abstract

Previous studies have indicated a link between neutrophil to lymphocyte ratio (NLR) and impaired fasting glucose (IFG), but the findings have been disputed. By conducting a real-world follow-up study, we can monitor the development of diseases and confirm the connection between NLR and IFG. A total of 1168 patients without IFG or T2DM were followed up for six years. At baseline, participants' NLR levels, fasting plasma glucose and other clinical characteristics were recorded. During the follow-up period, NLR levels and the prevalence of IFG were recorded. Ultimately, 45 individuals were lost to follow-up, leaving 1,123 participants for analysis. Using Group-Based Trajectory Modeling (GBTM), the sample was divided into three groups. The prevalence of IFG in the three groups was 12.1%, 19.4%, and 20.85%, respectively. Compared with the low-level NLR group, the hazard ratio of IFG in the moderate-level NLR group and high-level NLR group were 1.628 (1.109–2.390) and 1.575 (1.001–2.497), respectively. There was a significant interaction effect of BMI and NLR on the risk of IFG (P < 0.001). In this real-world follow-up study, we observed a positive association between NLR and the risk of IFG, with this relationship being exacerbated by obesity status.

## Introduction

Newly released data reported that approximately 720 million individuals were affected by prediabetes worldwide in 2021, and this number may increase to 1 billion by 2045^1^. In China, the prevalence of prediabetes was 19% in adults with age above 18 years old, and approximately 5% ~ 10% of people with prediabetes progress to having diabetes each year^2,3^. Prediabetes included three types, impaired fasting glucose (IFG), impaired glucose tolerance (IGT) and IFG combined with IGT. In this present study, we aimed to investigate the risk factor of IFG. IFG is an intermediate progression that fasting plasma glucose (FPG) levels were higher than normal but not yet high enough to be diagnosed as type 2 diabetes mellitus (T2DM) , that was first described in 1979^4^.

Previous studies reported that several factors can influence the levels of FPG, including obesity^5^, hypertension^6^, an unhealthy life style^7^, and dysimmunity^8^. The immune system, comprising both the innate and adaptive components, has been unequivocally linked to metabolic syndrome^9^. Specifically, innate immune activation, triggered by inflammation induced by obesity, has emerged as a key factor in its relation to insulin resistance^10^. Recently, one index of innate immune, Neutrophil to lymphocyte ratio (NLR), was attracted extensive attention in diabetes and its complications. A meta-analysis, including 11 studies, found that compared with control, the women with gestational diabetes mellitus have a higher NLR levels^11^. Winter Lauren et al.^12^ systematically reviewed the association between NLR and diabetic kidney, and their findings unequivocally demonstrated that individuals exhibiting elevated NLR levels exhibited significantly poorer kidney function. However, as an inflammation index of innate immune, it is not known whether NLR can predict diabetes mellitus in early stage, such as prediabetes progression.

Meanwhile, it is well known that obesity is the risk factor of IFG, and obesity-induce inflammation increased the level of NLR, but few studies were to explore the role of body mass index (BMI), an obese index, on the relationship between NLR and IFG. In this present study, we have followed up 1,168 participants in seven years, and detailed recorded their FPG, BMI and IFG status. Using the Group-Based Trajectory Modeling (GBTM), we have explored the prevalence of IFG in different NLR trajectory groups, and assessed the role of BMI in on the relationship between NLR and IFG.

## Methods

### Participants

**Figure 1:**
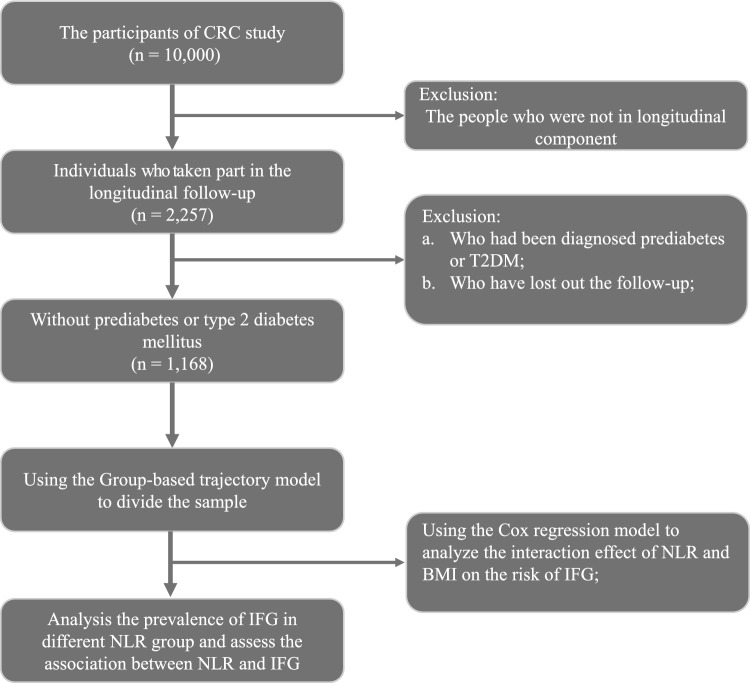
The flowchart of this study.

This study represents a longitudinal extension of our previous cross-sectional study, the Cardiometabolic Risk in Chinese study (CRC), which focuses on metabolic diseases in northern China. The CRC study initially involved a total of 10,000 participants at baseline, with 2,257 individuals participating in the longitudinal component. This subset of participants belongs to the same community and shares comparable research backgrounds, lifestyle habits, and cultural practices. Starting from 2012, we included this subset of individuals in the baseline assessment and conducted annual follow-ups encompassing physical measurements, serum testing, and questionnaires. The inclusion criteria were as follows: a) individuals were not diagnosed with T2DM or prediabetes; b) individuals completed the baseline test. The exclusion criteria were as follows: (a) individuals suffering from severe heart disease, kidney failure, lung failure, and other malignant tumors; (b) individuals with mental disorders unable to communicate normally. Finally, we specifically selected 1,168 patients as the study population (Fig. 1). All individuals have signed informed consent forms. This study has been approved by the Ethics Committee of Xuzhou Central Hospital.

### Clinical characteristics of participants

After overnight fasting (over 8 h), venous blood samples were collected from individuals, then those samples were separated by centrifugation for 15 min at 3000 rpm (1200 × g). Using an auto-analyzer (Type 7600, Hitachi Ltd. Tokyo, Japan), the levels of white blood cell counts (WBC), red blood cell counts (RBC), hemoglobin (HBG), hematokrit (HCT), blood platelet (PLT), Neutrophil, Lymphocyte, glutamic oxalacetic transaminase (AST), glutamic-pyruvic transaminase (ALT), total protein (TP), serum albumin (ALB), gamma-glutamyl transpeptidase (GGT), total cholesterol (TC), triacylglycerol (TG), high density lipoprotein (HDL), low density lipoprotein (LDL) and fasting plasma glucose (FPG) were determined in the central laboratory. All participants conducted the oral glucose tolerance test (OGTT) to detect the postprandial blood glucose.

Questionnaire and physical examination were carried out by the trained medical workers. Weight, height, systolic blood pressure (SBP) and diastolic blood pressure (DBP) of participants were measured and recorded. Participants should remove their shoes and stand on the height and weight measuring device for accurate height and weight measurements. BMI was assessed by weight and height, and the formula was as follow: BMI (kg/m2) = weight (kg) / height (m2). From 2012 to 2017, the levels of FPG and BMI were collected by every year.

### Outcome event and risk factors

In this study, the diagnostic criteria for prediabetes were based on the expert consensus documents of China^13^. The diagnostic criteria for IFG were as follows: FPG values between 5.6 mmol/L and 6.9 mmol/L. Once an individual was diagnosed with prediabetes, he/she would be excluded from the follow-up cohort and their onset outcome would be recorded. NLR was assessed by Neutrophil and Lymphocyte, the formula was as follow: NLR (%) = Neutrophil/Lymphocyte.

### Statistical analysis

R version 4.2.2(https://cran.r-project.org/) and STATA version 16.0(https://www.stata.com/stata16/) software were used to analyze the final data. Group-based trajectory model (GBTM) was performed to identify classes within a population that exhibit similar development trajectories. GBTM is a statistical method used to analyze the trajectories of continuous variables. GBTM can categorize data into various trajectory profiles using annual consecutive records of NLR and BMI levels. According to the NLR development trajectories, which were defined by GBTM, our data was divided into three groups. The differences of clinical characteristic between groups were analyzed using ANOVA and the chisq.test. To explore the interactive role of BMI on the relationship between NLR and prediabetes, the development trajectories of BMI were assessed by GBTM. Using the Cox proportional hazard model and time-event curve, we have analyzed the interaction effect of NLR and BMI on the prevalence of IFG, and compared the prevalence of prediabetes in different subgroups. A two-sided P value of less than 0.05 was considered statistically significant.

### Ethics approval and consent to participate with methods declaration

The study was reviewed and approved by the ethics committee of the Xuzhou central hospital. The NO. of ethics committee approval is XZXY-LJ-20201110–060. The research and statistical methods used in this study are all in accordance with the relevant guidelines and regulations.

### Statement

This article has been obtained informed consent from all subjects and legal guardians.

## Results

In this present study, a total of 1,123 participants were recruited the finally analysis, with age 47.70 ± 10.39 year-old, 40.2% (n = 451) of males, mean follow-up time of 3.54 years and 7947.5 person-years of observations. At the baseline, the FPG was 5.31 ± 1.23 mmol/L, NLR was 1.80 ± 0.71%, and no patient with pre-diabetes was observed.

**Figure 2:**
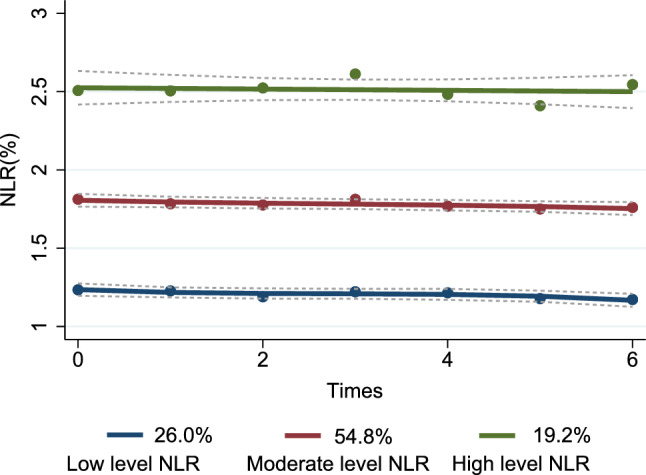
The trajectories of long-term normal NLR by GBTM.

In order to determine the optimal number of subgroups, we conducted a comparison of BICs obtained through GBTM and found that the optimal number of subgroups was three (Fig. 2): Low level NLR group (n = 282, 26.0%), Moderate level NLR (n = 629, 54.8%), and High level NLR (n = 212, 19.2%).

The baseline characteristics, according to the trajectories of NLR, are presented in Table1. Although most of indices were the same in the three groups, some significant differences of levels of WBC, FPG, AST, Neutrophil counts, Lymphocyte counts, TP, GGT and the smoking status were observed.

**Table 1:** The clinical characteristic of participants according to the trajectories of NLR.

	NLR (%)			P
	Low level NLR	Moderate level NLR	High level NLR	
n	281	629	212	
Sex (male)	103(36.5%)	251(39.9%)	97(45.8%)	0.115
BMI (kg/m^2^)	23.86 ± 3.14	23.91 ± 3.02	23.78 ± 3.16	0.933
Age (years)	47.79 ± 10.51	47.55 ± 10.10	48.02 ± 11.60	0.284
WBC (× 10^9^/L)	5.63 ± 1.42	6.07 ± 1.47	6.36 ± 1.61	< 0.001
RBC (× 10^12^/L)	4.63 ± 0.43	4.67 ± 0.45	4.69 ± 0.48	0.473
HBG (g/L)	137.17 ± 15.08	137.79 ± 15.10	138.65 ± 14.98	0.558
HCT (%)	41.45 ± 3.96	41.94 ± 4.36	41.61 ± 4.02	0.68
PLT (× 10^9^/L)	215.17 ± 43.80	209.18 ± 46.49	212.48 ± 43.91	0.363
AST (U/L)	17.00(15.00 ~ 21.00)	17.00(15.00 ~ 20.00)	17.00(14.00 ~ 19.00)	0.011
ALT (U/L)	17.00(12.00 ~ 23.00)	16.00(12.00 ~ 23.00)	16.00(12.00 ~ 22.00)	0.656
Neutrophil (× 10^9^/L)	2.69 ± 0.73	3.60 ± 1.05	4.18 ± 1.32	< 0.001
Lymphocyte (× 10^9^/L)	2.43 ± 0.62	2.14 ± 0.48	1.96 ± 0.46	< 0.001
TP (g/L)	76.27 ± 4.33	75.70 ± 4.21	75.45 ± 4.15	0.039
ALB (g/L)	45.58 ± 1.95	45.58 ± 1.83	45.51 ± 1.51	0.218
GGT (umol/L)	20.00(14.00 ~ 29.00)	21.00(14.00 ~ 34.50)	18.00(14.00 ~ 26.00)	0.047
FPG (mmol/L)	5.17 ± 0.65	5.36 ± 1.23	5.37 ± 1.10	0.037
TC (mmol/L)	4.98 ± 0.78	4.94 ± 0.90	4.79 ± 0.81	0.031
TG (mmol/L)	1.17(0.81 ~ 1.69)	1.17(0.80 ~ 1.92)	1.19(0.80 ~ 1.79)	0.845
HDL (mmol/L)	1.52 ± 0.38	1.49 ± 0.38	1.45 ± 0.36	0.181
LDL (mmol/L)	3.04 ± 0.68	2.97 ± 0.78	2.89 ± 0.69	0.152
SBP (mmHg)	123.39 ± 16.30	124.04 ± 16.20	122.88 ± 16.80	0.776
DBP (mmHg)	78.52 ± 10.11	79.31 ± 10.62	77.43 ± 9.73	0.127
Smoking				
Yes	56(19.9%)	174(27.7%)	69(32.6%)	0.005
No	225(80.1%)	455(72.3%)	143(67.4%)	
Drinking				
Yes	97(34.5%)	219(34.8%)	76(35.8%)	0.935
No	184(65.5%)	410(65.2%)	135(64.2%)	

*NLR* Neutrophil to lymphocyte ratio, *BMI* body mass index, *WBC* white blood cell count, *RBC* red blood cell count, *HBG* hemoglobin, *HCT* hematocrit, *PLT* platelet count, *AST* glutamic oxalacetic transaminase, *ALT* glutamic-pyruvic transaminase, *TP* total protein, *ALB* albumin, *GGT* gamma-glutamyl transpeptidase, *FPG* fasting plasma glucose, *TC* total cholesterol, *TG* triglyceride, *HDL* high density lipoprotein, *LDL* low density lipoprotein, *SBP* systolic blood pressure, *DBP* Diastolic blood pressure.

The association between NLR and the developing risk of prediabetes. Table 2 showed the hazard risk (HR) of prediabetes with different groups. In this table, the prevalence of prediabetes in low level NLR group was 12.1% (34/281), that was lower than moderate level NLR group (19.4%) and high level NLR group (20.8%). Treading the low-level NLR group as the reference, the COX regression showed that the crude risk for the moderate-level NLR group was 1.669 (1.140 ~ 2.443), and high level NLR was 1.626(1.027 ~ 2.575). After adjustment for other potential characteristic including sex, baseline age, baseline BMI, baseline TC, baseline TG, baseline SBP, baseline DBP, baseline AST, baseline ALT, smoking status and drinking status, moderate level NLR group still positively associated with the risk of developing prediabetes (1.628, 95% C.I 1.109 ~ 2.390). In addition, the trend test was implemented to analyze the linear association between NLR level and the risk of developing prediabetes. The results showed that even if adjusting for other potential factors, there was a positively association in the model. In the stratified analysis for subgroups, we found that the prevalences of IFG showed no significant difference among different subgroups (Supplement Fig. 1).

**Table 2:** Cox regression model to analyze the association between NLR levels and the risk of prediabetes.

	Non-prediabetes	Prediabetes	Model1		Model2	
			HR	95%C.I	HR	95%C.I
Low level NLR	248(87.9%)	34(12.1%)	1	1	1	1
Moderate level NLR	507(80.6%)	122(19.4%)	1.669	1.140 ~ 2.443	1.628	1.109 ~ 2.390
High level NLR	168(79.2%)	44(20.8%)	1.626	1.027 ~ 2.575	1.575	1.001 ~ 2.497
P for trend	0.008		0.032		0.047	

Model1: adjustment for sex, age and BMI.

Model2: adjustment for sex, age(baseline),BMI(baseline), TC(baseline), TG(baseline), SBP(baseline), DBP(baseline), AST(baseline), ALT(baseline), smoking and drinking.

**Figure 3:**
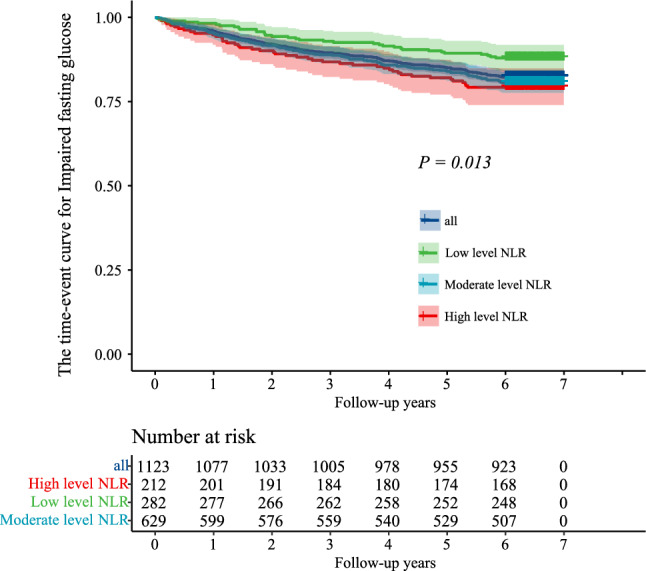
The time-event curve for the risk of developing prediabetes in different groups.

3.4 The time-event curve for the risk of developing prediabetes in different groups. Figure 3 showed the time-event curve for the risk of developing prediabetes in three groups. After seven years follow-up, a total of 34 (12.1%) individuals in the low level NLR group developed prediabetes, 122 (19.4%) individuals in the moderate level NLR group developed prediabetes, and 44 (20.8%) individuals in the high level NLR group developed prediabetes. There was a significant difference among three groups, and the P value was 0.013.

The interaction effect of BMI on the association between NLR and IFG.

**Figure 4:**
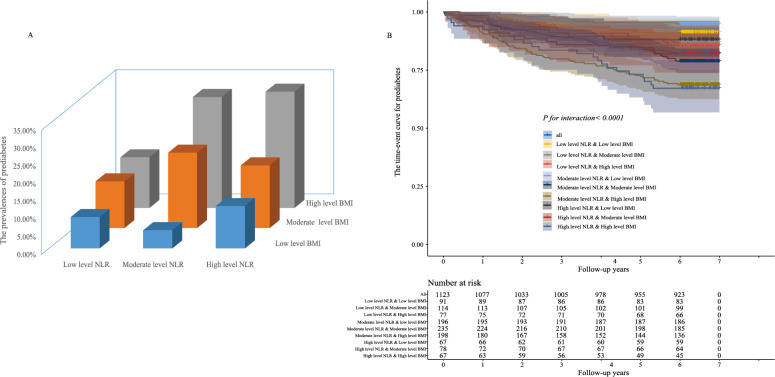
The prevalence of IFG in different subgroups.

To the best of our knowledge, there is a close association between obesity and the risk of developing IFG. In order to mitigate the impact of obesity, the adjusted Cox regression model (Table2) incorporated the baseline BMI. However, it should be noted that the BMI of individuals varied over the course of the follow-up period. Therefore, it is crucial to examine the role of BMI in the connection between NLR and IFG. Employing GBTM, we delineated the long-term patterns of normal BMI and identified an optimal subgroup to investigate its significance. In Fig. 4A, we presented the prevalence of IFG in various subgroups and observed that the highest prevalence was found in the subgroup characterized by high levels of NLR and BMI. The time-event curve demonstrated that the association between NLR and IFG was modified by BMI (P < 0.001, Fig. 4B). Moreover, individuals with high levels of NLR and BMI exhibited an increased risk of developing prediabetes.

## Discussion

In this present study, the participants were classified into three categories based on their NLR trajectories over a period of seven years: the low level NLR group, the moderate level NLR group, and the high level NLR group. The prevalence of IFG in three groups were 12.1%,19.4% and 20.85%, respectively. Further analysis explored the interaction effect of BMI on the relationship between NLR and IFG. The results revealed a significant effect, indicating that individuals with both high BMI and high NLR exhibited the highest prevalence of IFG, reaching 32.8%.

Some previous studies have reported the association between NLR and IFG, but a controversial conclusion was reached on this matter. JK Kim et al.^14^ found that there was a significantly negative relationship between NLR and FPG in normal populations, and in individuals with IFG, there was no significant association. However, in a Chinese cohort study, CJ Zhang^15^ reported that larger increase of NLR could increase T2DM risk, and there was a positive relationship between NLR and the risk of T2DM. In our study, we discovered that individuals with elevated NLR had a higher incidence of IFG, indicating a positive correlation between NLR and IFG risk. This follow-up study confirmed the previous finding that NLR is associated with an increased risk of both FPG and IFG.

It is well known that chronic systemic inflammation plays a key role in the mechanism of metabolic diseases^16,17^. NLR, assessed by neutrophils and lymphocytes, is considered a cost-effective index to reflect the status of systemic inflammation^18^. For instances, a Chinese large sample study (n = 90,237) found that NLR was related to the prevalence of T2DM in men and women^19^; T Akase et al.^20^ reported that NLR might be a useful biomarker for diabetic kidney disease. A systematic review and meta-analysis found that NLR was positive related with glycated hemoglobin levels^21^. In our study, we found that elevated NLR was positive associated with FPG and increased the prevalence of IFG. This result was agreed with previous researches. The NLR increasing FPG levels and the associated risk of IFG may be linked to heightened insulin resistance. Animal studies have revealed that neutrophil elastase in adipocytes can diminish insulin receptor substrate 1 and inhibit insulin-induced AKt protein kinase phosphorylation, consequently elevating insulin resistance levels^22^. A study conducted in Turkey found that NLR significantly increased only among obese patients with insulin resistance^23^. In overweight/obese school children^24^, NLR was also identified as a risk factor for insulin resistance. It is well-established that being overweight or obese is a risk factor for IFG^25^. However, few studies have explored the interaction effect of overweight and NLR on IFG. The findings of our study suggest that the prevalence of IFG is higher in the group with both higher BMI and NLR levels compared to those with only higher BMI or NLR levels. This result confirms that there is an interaction effect of BMI and NLR on the risk of IFG. BMI, which is determined based on weight and height, is used to indicate the presence of obesity^26^. Elevated BMI level is the primary criterion for diagnosing obesity^27^. Research has indicated that obesity can trigger persistent inflammation in the body, leading to insulin resistance^28^. Chronic inflammation in the body can give rise to metabolic disorders. A comprehensive book that reviewed the pathophysiology of obesity found that obesity has inflammatory components directly and indirectly related to major chronic diseases such as diabetes, atherosclerosis, hypertension, and several types of cancer^29^. In our study, we discovered that increased BMI exacerbated the relationship between NLR and the risk of IFG. This interaction effect was validated using the GBTM method. GBTM is an innovative technology for analyzing longitudinal data variations. In comparison to cross-sectional data, longitudinal data is more adept at capturing fluctuations in the target variable. These dynamically changing target variables are more indicative of real-world outcomes.

Although our study was a follow-up research, it is important to elucidate the association between NLR and the risk of IFG from a pathological perspective. An elevated NLR may activate inflammatory pathways and cascades by activating corresponding receptors and blocking insulin receptor signaling, consequently leading to insulin resistance and T2DM^30^. The widely accepted theory about chronic inflammation and diabetes is that the JNK/IKK NF-κB pathway is activated, and both JNK and IKK are thought to be the mediators of insulin resistance induced by inflammation^31–33^. They exert their action by phosphorylating the serine residues on insulin receptor substrates (IRS) proteins, thereby blocking the phosphorylation of IRS on tyrosine residues subsequent to activation by the insulin receptor^34^. The ensuing insulin resistance is a primary clinical feature of IFG, T2DM^35^, and other related diseases, and is also one of the main reasons for the occurrence of T2DM in obese populations^36^.

There were some limitations in this study. Firstly, this is the follow-up study in real-world, and the results need to verify in experiment research. Secondly, the participants of this study were from North China, this epidemiological result need to verify in other country and ethnic/Race.

In summary, our study used GBTM analysis to confirm a positive association between NLR and the risk of IFG in the follow-up population. We also found that obesity exacerbates this relationship, resulting in the highest prevalence of IFG in individuals with higher NLR and obesity. Our findings suggested that public health researchers should prioritize their focus on community populations with persistent increases in NLR and BMI during routine disease prevention efforts, aiming to decrease the incidence of diabetes.

### Supplementary Information

Supplementary Information 1.

Supplementary Information 2.

## Data Availability

All data generated or analyzed during this study are included in this manuscript.
