# Supplementary figures and images for: Follow-up study to explore the relationship between Neutrophil to lymphocyte ratio and impaired fasting glucose—using the group-based trajectory modeling
Source: Sci Rep. 2024 Jun 18;14:14064. doi: 10.1038/s41598-024-64701-5 (PMC11189411; doi:10.1038/s41598-024-64701-5)

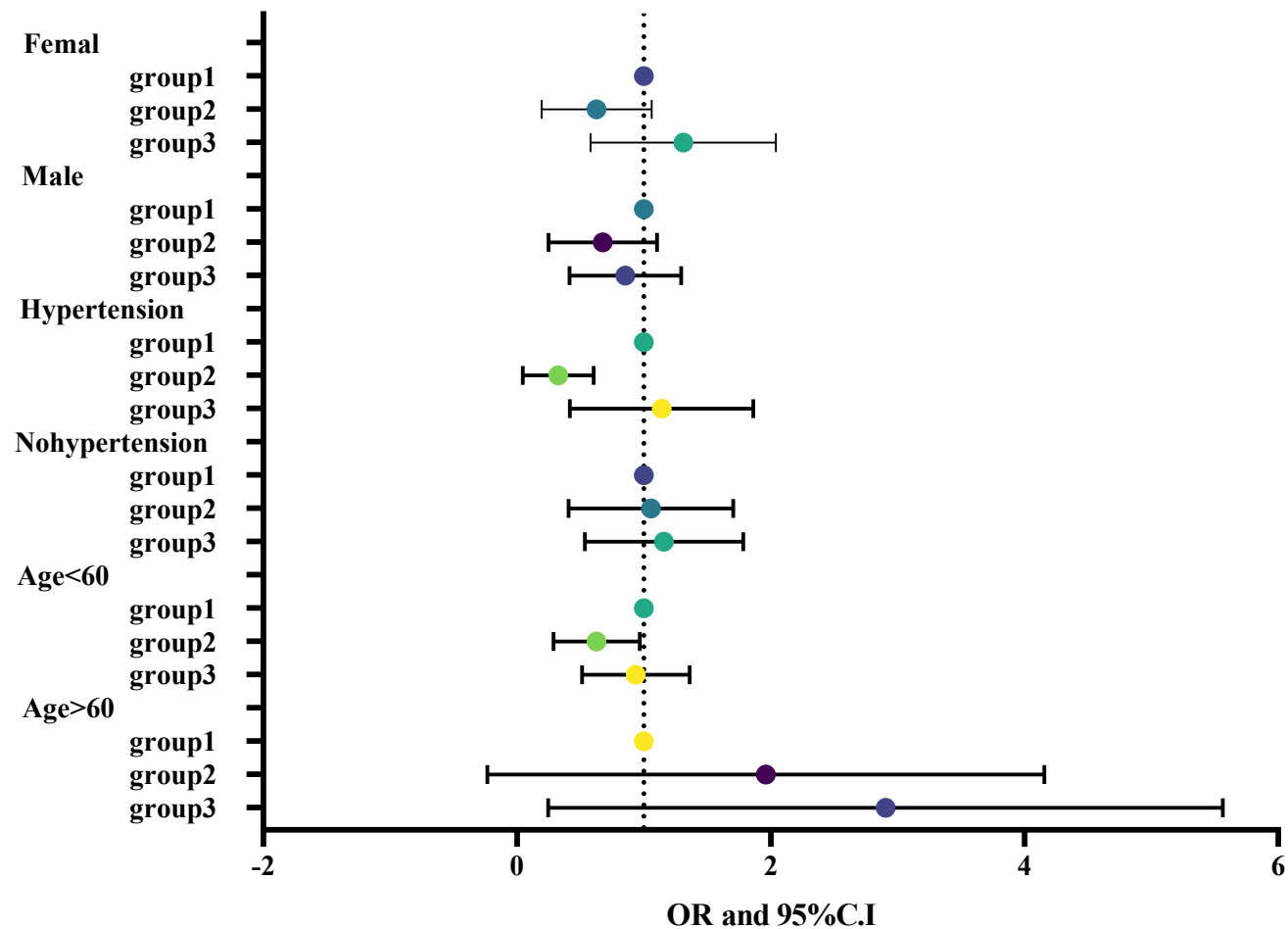

Supplement: Supplementary file 1 — Supplementary Information 1. [file 41598_2024_64701_MOESM1_ESM.pdf]

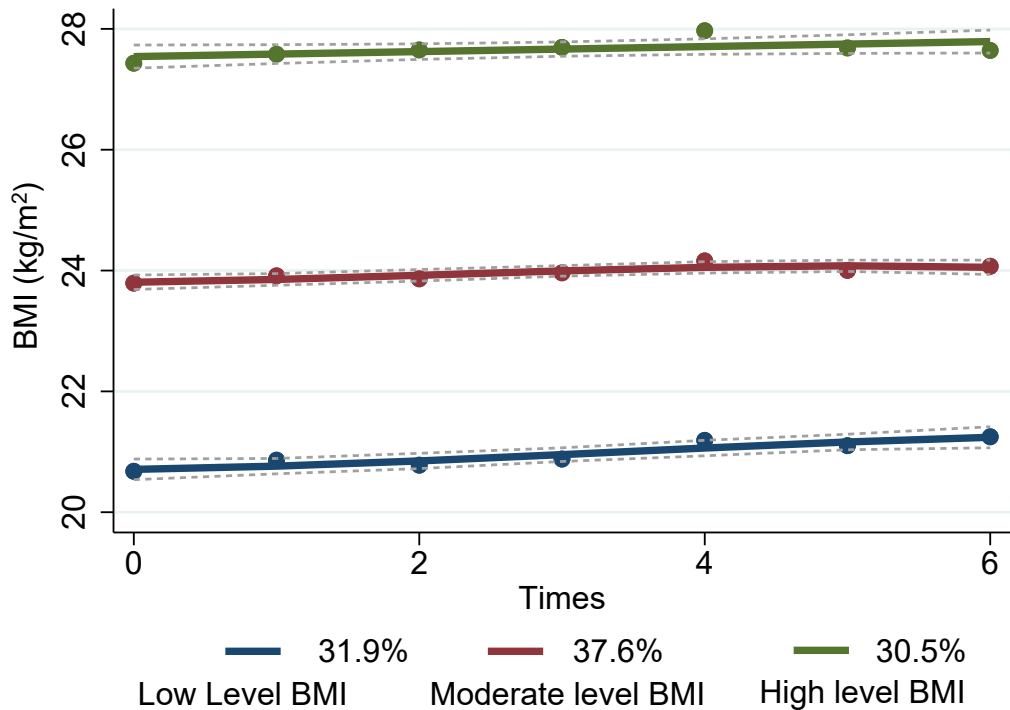

Supplement: Supplementary file 2 — Supplementary Information 2. [file 41598_2024_64701_MOESM2_ESM.pdf]
